# Burnout of school principals in Poland: work demands, resources, and stressors

**DOI:** 10.3389/fpubh.2025.1610378

**Published:** 2025-07-22

**Authors:** Joanna Kołodziejczyk, Grzegorz Mazurkiewicz, Jakub Kołodziejczyk

**Affiliations:** Institute of Public Affairs, Faculty of Management and Social Communication, Jagiellonian University, Kraków, Poland

**Keywords:** burnout, extra-organizational stressors, job resources, Oldenburg Burnout Inventory, burnout in school principals

## Abstract

**Background:**

School principals face increasing professional challenges and psychological strain, amplified by extra-organizational stressors such as political and systemic changes, climate change, war, and economic instability. These factors can elevate job demands and increase the risk of professional burnout.

**Methods:**

This study examined the relationship between burnout among 117 Polish public school principals and their subjective perception of extra-organizational stressors as well as the use of job resources. Burnout was measured using the Oldenburg Burnout Inventory, while support sources and perceived external pressures were assessed via a custom questionnaire. Multiple regression analyses were conducted to assess predictive relationships.

**Results:**

Burnout was significantly associated with perceptions of changes in the educational system. Institutional support from the education system was linked to lower burnout levels, while support via social media was associated with higher burnout and disengagement. Other factors, such as concerns about the economy, climate change, and war, were not significantly associated with burnout.

**Conclusion:**

Findings highlight the critical impact of systemic educational reform as a unique stressor contributing to burnout among school principals. Institutional support appears to buffer this effect, while reliance on informal networks such as social media may reflect deeper professional distress. These results suggest the need for structural support mechanisms tailored to school leadership roles.

## 1 Introduction

School principals around the world are working under enormous pressure. They are often seen as responsible for the mental health and wellbeing of their staff, and in their roles as advisory bodies and problem solvers for teachers, they sometimes carry emotional burdens of their personal and professional lives. Of course, they are also responsible for the quality of the school's work and its image in the community, but above all for the struggles, stress and trauma of students, as well as for meeting their social and emotional needs. School principals are also accountable for academic achievements of their students and for wellbeing of their teachers and other staff. As a result, principals absorb experiences and irritations of both students and staff, and in many cases hear complaints and concerns directly from parents and community members. The role of school principal can be both demanding and isolating and it may contribute to high levels of stress.

A representative study conducted in 2022 in the United States by the RAND Corporation found that 85 percent of school principals experienced work-related stress, 48 percent struggled with burnout, and 28 percent reported symptoms of depression (1).

These are disturbing data. Unfortunately, we do not have knowledge about the current mental condition of school principals in Poland. However, it is known that school principals in Poland work under similar pressure. Recent years have brought global and regional crises, frustrations and various challenges. A few important examples include, first of all, local crises, i.e., the widely criticized reform of the Polish educational system (2) and related processes in 2015–2023 and the teachers' strike in 2019, as well as global crises, i.e., the increasingly dangerous ecological and climate crisis, the pandemic COVID-19, war in Ukraine or economic disturbances. The beginning of 2025 does not bring promises of improvement, and in Poland we are struggling with a new challenge related to the election results on October 15, 2023, the emergence of a new government and new ideas for Polish education, and the resulting need to introduce changes and to reform schools and the education system (which is a necessary and desirable although extremely complicated process). Those pressures create extremely difficult working conditions for school principals. Do school principals have enough resources to cope with that?

Among many challenges school principals face, those that mental health related (staff burnout, student anxiety, or others) are becoming increasingly important. The latest PISA 2022 research report revealed low socio-emotional competences of Polish 15-year-olds. The level of curiosity, cooperation, empathy, assertiveness and stress resistance of Polish teenagers is definitely below the average achieved by respondents from other countries, and when it comes to perseverance and emotional control, our students took last place. This seems worrying (3).

It is expected that school principals should provide support for both groups: students and teachers. Students are in deep emotional crisis. There is also disturbing information about teachers' job satisfaction. Teachers are exposed to the threat of professional burnout. International research shows that the sense of job satisfaction reduces the risk of burnout resulting in leaving their jobs. Job satisfaction has a positive impact on teachers' motivation to work and their involvement in working with students. The PIRLS study shows a decrease in the declared sense of professional satisfaction felt by teachers of students participating in the study, compared to the previous edition of the study in 2016. In the countries participating in the PIRLS study, almost all students had teachers with high and average levels of job satisfaction (61% and 32%, respectively); only 7% of children were taught by teachers declaring low levels of job satisfaction. The result from Poland stands out negatively compared to other countries. Taking into account the level of professional satisfaction, Poland occupied the last place. In the PIRLS 2016 study, Poland also took a distant, although relatively better, place (40 out of 50 countries) on the scale of teachers' professional satisfaction. Similar results (the penultimate place in terms of professional satisfaction in 2019, worse results compared to the previous edition) were also observed in the TIMSS 2019 study in relation to science and mathematics teachers. The data confirm the downward trend in teachers' job satisfaction and, simultaneously, show that this is an important challenge to be faced by the Polish education system (4).

Unfortunately, negative emotions impact the process of hiring teachers. There are predictions that there is a deficit of ~25 thousand teachers in the Polish educational system and there is no perspective for elimination of all the shortcomings. Low salaries mean that people who graduate are not willing to pursue the teaching profession. Lack of the prestige of the profession decreases the number of people willing to work as teachers (5).

To summarize the description of the context: school principals need many specific skills, but above all they need support and a sense of purpose. Unfortunately, school principals are often left to fend for themselves, responsible for securing both financial and pedagogical support at a time when challenges, frustrations, and setbacks are steadily mounting. They may shoulder many duties yet have few avenues for meaningful assistance.

The importance of educational leadership as an influence on student learning is clear. Effective school leadership leads to school effectiveness, which in turn leads to improved student outcomes (6).

### 1.1 Literature review. Professional burnout

Since the 1970s, the problem of burnout has aroused great interest among researchers, which has intensified in recent decades due to the development of the concept of psychosocial wellbeing in the professional context. The concept of burnout syndrome is used in the literature to describe a set of mental and somatic symptoms resulting from chronic stress related to an important and permanent job for a given person (7).

It is worth noting that there are no strict criteria that would allow for the diagnosis of whether a given person is or is not “burnt out.” One can only assess the severity of symptoms and conclude about their greater or lesser intensity. Burnout syndrome is therefore considered as a continuum of symptoms—from the state of complete health to complete “burnout” (7). According to Christina Maslach, an American researcher, symptoms of burnout syndrome include emotional exhaustion, depersonalization and a reduced sense of personal achievement (8). Other researchers draw on Maslach's concept. The World Health Organization, for example, recognizes burnout as a syndrome that is a psychological reaction to chronic stress resulting from professional workload, the characteristic features of which are exhaustion, mental distance from work, cynicism, and lower professional effectiveness (9, 10). Burnout is a destructive phenomenon that negatively affects mental and physical health of the person affected by it. It also has its economic and social dimensions (7, 11). Therefore, burnout is a significant challenge to be dealt with while managing organizations in such a way as to minimize the possibility of its occurrence.

Burnout syndrome can potentially affect every working person. Those who while performing their work, enter into numerous and sometimes difficult interpersonal relationships with others, for example health care workers or education workers are particularly vulnerable (12).

The issue of burnout in the group of education workers has been repeatedly addressed in research conducted in Poland in recent years (10). Teachers at various levels of education, from primary teachers to university teachers were the most frequently involved in surveys. Some of the studies focused on teachers of particular subjects, e.g., physical education (12), religion (13), as well as other groups of school employees including school teachers (14) and school psychologists (15). School principals as a group of respondents were involved in only one study (16) as one of three groups (also including teachers and pedagogues). More often, school principals as a professional group are the subject of research in other contexts related to burnout, e.g., professional and psychosocial wellbeing (17), requirements and workload of the professional role (18, 19).

With regard to the work of school principals, it is noted that the role uniquely exemplifies a social profession, more specifically, a helping profession (or, equivalently, a human-oriented profession) Pyzalski (18). In addition to the professional requirements characterizing the helping profession, the principal is also responsible for the coordination and quality of activities of all staff (18). The key to the work performed by the principal in the context of the helping profession is, in addition to professional knowledge, who they are as a person, their temperament, personality, education, current and past personal experiences. Personality becomes the principal's working tool. Another characteristic feature of this work refers to interpenetration of professional, personal and private spheres, and to impossibility of fully separating them all. This is due, among other things, to the enormous burden of responsibilities and the inability to complete all tasks within the scheduled working time at school. Such a situation may translate into disruptions in family relationships or cause health problems or health-promoting behaviors. It is worth building a principal's resilience, as resilient leaders leverage mental agility, emotional stability, social acumen, ethical conviction, and physical stamina to navigate complexity and lead their schools effectively (20).

Another important aspect of the principal's work is permanent communication with various entities—including teachers and other employees, students, parents, and institutions—which involves diverse topics, is often emotionally demanding, and requires principals to maintain full control over their own emotions (18). The key feature of the helping professions also includes imbalance between “giving,” i.e., what the principal puts into work (energy, time, commitment) and the results obtained, i.e., “taking.” An additional challenge for this role is sometimes attributed to circumstances of decision-making (under pressure, immediately, in a crisis situation). Those characteristics are united by the category of responsibility understood in various ways—in the legal, personal and psychological contexts, additionally expanded to include responsibility for actions undertaken by all employees led by the principal in question. Often, the principal, feeling responsible but also convinced that only they are able to cope with the challenges, is unable or unwilling to delegate tasks, which causes additional difficulties in coping with their implementation. Moreover, actions and decisions taken by the principal are public, and they are subject to social evaluation. Therefore, the requirements associated with performing the role of a principal are very high, and it is difficult to meet them. This role involves an overload of responsibilities and information, as well as a significant emotional burden (21). The inability to cope with responsibilities and the lack of protective factors lead to professional stress, and prolonged stress may turn into burnout (18).

Burnout is mainly a state of mental, emotional and physical exhaustion experienced by school principals as a result of long-term work-related stress. This stress can come from a variety of sources, and as a result, many executives find it difficult to reconcile demands of their jobs with their own wellbeing, leading not only to burnout but also, in some cases, to leaving their professions.

In the field of education in Poland, research primarily employs two versions of the Maslach Burnout Inventory (MBI and MBI-GS) for examining burnout. The OLBI (Oldenburg Burnout Inventory) was used in one study in Poland (22). The OLBI questionnaire is the result of a different approach to conceptualizing burnout. Demerouti et al. (23) proposed understanding of burnout based on the Job Demands–Resources model (JD-R). This model assumes the interaction of two processes. The former is related to the fact that each profession has specific requirements relating to the physical, social and organizational aspects of work. In this process, the demanding aspects of work lead to overload and, in the long run, to burnout (exhaustion). The latter process is related to activation of resources to cope with work demands and engage one's own physical and mental effort. The lack of appropriate resources makes it difficult or impossible to meet job demands, which leads to withdrawal behavior and, in the long term, to withdrawal from work.

Work (occupational) demands refer to the physical, psychological, social, or organizational aspects of work that require both physical and mental (cognitive and emotional) effort and are associated with costs incurred by employees (occupational stressors) (24). Based on a literature review, three groups of factors related to work requirements, which are important for practitioners, were identified (25). These include qualitative work demands (emotional, mental, physical, work-home conflict), quantitative work demands (work under-load and overload, pace of change), and organizational work demands (negative changes, bureaucracy, harm, role conflict, interpersonal conflicts). Researchers most often focus on the influence of factors related to the work context (such as those mentioned above), and less frequently examine non-organizational (extra-organizational) factors that may similarly influence the experience of stress and burnout (26, 27).

Job resources are defined as aspects that enable achievement of professional goals, reduce professional demands and associated physiological and psychological costs, and that stimulate learning and personal development (24). Job resources can be located at the following various levels (24): organizational (e.g., salary, career opportunities, job security), interpersonal (e.g., support from superiors and co-workers, team climate), position (e.g., role clarity, participation in decision making) and at the task level (e.g., skill diversity, task identity, task importance, autonomy and performance feedback). Work resources may have a mitigating effect on job requirements, such as professional autonomy, social support, the quality of relationships with superiors and feedback on performance (28), and may also play a protective role toward other resources.

### 1.2 Extra-organizational stressors

Extra-organizational stressors are defined as environmental factors unrelated to work that can negatively impact employees (27) and are beyond the influence of the organization and its management (29). Among this group of stressors, Ivancevich and Matteson (30) identified societal or technological changes, family relocation, and economic and financial conditions. Additionally, Michie (31) highlights family problems and life crises. Other stress-inducing factors may include the fear of terrorism, which can reduce work performance (32), as well as the spread of infectious diseases such as SARS and COVID-19, which have had a strong negative impact on employee wellbeing (33, 34). The significance of extra-organizational stressors stems from their influence through a feedback loop between work and the external environment: problems outside of work affect individuals in the workplace, which in turn exacerbates issues outside of work (35).

Four, currently important, different extra-organizational stressors were selected to this study. There are multiple voices underlying how the constant change of the educational policies and practice (36), caused by Polish political changes and global reform movement, impact the quality of work of the educators and their level of stress. Overwhelming, global warming problem (37) not only changes the conditions of life for whole societies but also influences the world of education on different levels: changes learning environments, content and methods of teaching, aims of the education and raises fear and anxiety. War just behind Polish border had change the reality of Polish schools pushing forward issues that were even not considered previously. Physical and psychological safety, logistic issues connected to the consequences of the war never analyzed since fifties, the presence of thousands traumatized students from Ukraine and more other issues became incredible burden for Polish school principals (38). And finally, due to global processes, pandemic, war and economical sanctions on Russia various economical phenomena forced the economic crises (39) influencing the financial safety of schools. Taking into account the contextual burdens, stress levels and enormous expectations toward the entire educational system and schools in Poland related to planned changes resulting from political changes and the multiplicity of tasks facing global and national society, one can only expect an increase in the scope of principals' responsibilities. This professional group is already working in a situation of emotional strain and the risk of burnout.

The first aim of the study was to check the relationship between burnout, measured using the Oldenburg Burnout Inventory, and subjectively perceived extra-organizational stressors: (1) changes in the education system, (2) threats related to climate change, (3) economic crisis, (4) war, as factors increasing work demands. The second aim was to check in which areas of job resources, the principals participating in the study, most often look for support, i.e., which support factors actually play a role. Therefore, questions were asked about the degree of resource utilization (scale of use of the assistance) of: (1) school employees, (2) other principals, (3) educational system institutions, (4) support via social media. To achieve the study aims we stated two research questions. First: to what extend school principals feel pressure of the external stressors during their work. Second: to what extend school principals are using support of the resources accessible to them.

## 2 Methodology

### 2.1 Job burnout investigation

To measure burnout, the Oldenburg Burnout Questionnaire was used in the study. It is based on the assumptions of the Job Demands—Resources model (JD-R) and measures two dimensions of burnout: exhaustion and disengagement (lack of commitment to work/distance from work). Both scales reflect the two processes of the job demands-resources model. The adaptation and psychometric properties of the Polish version of the Oldenburg Burnout Questionnaire were published twice. In the first study, the respondents were people working in social service professions including teachers, medical staff, police officers (40), while in the second study, the respondents included nurses, social workers, teachers, as well as administrative workers, salespeople, professional soldiers and manual workers (41).

To measure burnout of principals of schools and institutions, the OLBI questionnaire in the Polish version was used (41). It measured two dimensions of the professional burnout: exhaustion and disengagement.

The questionnaire consisted of 16 items (8 in each subscale). Responses were collected on a four-point scale, where 1 meant “I strongly agree” and 4 meant “I strongly disagree.” Each subscale was a bipolar construct, containing four positively formulated items and four negatively formulated items. The unidirectionality of the scale was preserved by inverting negatively worded items. As a result of summing the ratings for each subscale item divided by their number (8), the result for each sub-scale was obtained. A higher score indicated a higher level of burnout for each of the sub-scales (exhaustion and engagement, respectively). The sub-scales were characterized by a satisfactory reliability coefficient, Cronbach's a for the disengagement sub-scale was 0.67, and for the exhaustion subscale it was 0.86, which means the consistency of the scales used.

### 2.2 Stressors investigation

When examining the area of requirements faced by principals at work, four extra-organizational stressors were taken into account. The first one referred to the subjective assessment of the direction of changes taking place in the education system, measured by the question “How do you assess the changes currently taking place in the education system?.” Responses were collected on a 5-point scale from 1—“they are heading in the right direction” to 5—“they are heading in the wrong direction” (To ensure consistency in interpretation, the scale was reversed prior to the analysis.). The next three stressors related to concerns related to information about climate change, economic crises and the threat of war. Responses were collected on a 5-point scale from 1—“I am not interested” to 5—“to a significant extent.”

### 2.3 Support investigation

In the group of the resources that can be used by principals looking for support, the following four aspects of social support were taken into account: help from school employees (vice principals, teachers, etc.), help from other principals, help from institutions of the education system (school management body, school board, etc.), help via social media (e.g., Facebook groups). Responses were collected on a 6-point scale from 1—“never” to 6—“very often.”

### 2.4 The study sample characteristics

The survey questionnaire was sent to all public school principals in a large city. One hundred twenty-six returns were received, representing almost 65% of working principals. After excluding questionnaires with missing significant data, 117 were qualified for the final analysis. The majority of the respondents were female (75.2%). The majority of principals participating in the study are serving their third or subsequent term as principals (41.7%). The surveyed group is dominated by primary schools (63.2%). Description of the characteristics of the study group can be found in Table 1).

**Table 1:** Characteristics of the study sample (*N* = 175).

**Descriptives**	**n**	**%**
**Gender**		
Female	88	75.2
Male	29	24.8
**Term in the position**		
1	48	41.0
2	28	23.9
3 or more	41	35.0
**Type of the school**		
Primary	74	63.2
School complex (primary and secondary school)	10	8.5
Secondary	33	28.2

### 2.5 Ethics approval

The study on the relationship between burnout among school principals in Poland and work demands, resources, and external stressors was approved by the Research Ethics Committee of the Faculty of Management and Social Communication at Jagiellonian University (KEBN/WZKS/UJ), No. 35/2024. The approved protocol complies with applicable legal regulations in Poland and best practices at Jagiellonian University in the field of social sciences and the discipline of management and quality science. Before the study commenced, informed consent was obtained from all participants.

## 3 Results

Descriptive statistics of the subscales of burnout and extra-organizational stressors and resources of school principals are provided in Table 2. The presented data show that the mean for the exhaustion scale was 2.66 (SD = 0.54), while for the commitment scale the mean was 2.32 (SD = 0.44). The burnout subscales, exhaustion and disengagement, are strongly positively correlated (*r* = 0.73).

**Table 2:** Descriptive statistics for subscale exhaustion and disengagement.

**Scale**	**N**	**Mean**	**SD**	**Min**.	**Max**.
Burnout	117	39.83	7.28	22.00	61.00
Exhaustion	117	21.25	4.34	9.00	31.00
Disengagement	117	18.58	3.48	11.00	30.00

In the group of the extra-organizational stressors included in the study (Table 3), the highest mean was achieved by the perception of the economic crisis (*M* = 4.09, SD = 0.91) and the threat of war (*M* = 4.13, SD = 0.92), and the lowest was the threat related to with climate change (*M* = 3.61, SD = 1.15). However, the assessment of changes taking place in the education system had an average of 4.08 (SD = 0.97).

**Table 3:** Descriptive statistics for the extra-organizational stressors.

**Extra-organizational stressors (workload)**	**N**	**Mean**	**SD**	**Min**.	**Max**.
Changes in the educational system	117	4.08	0.97	1.00	5.00
Climate change	116	3.63	1.15	1.00	5.00
Economic crisis	117	4.09	0.91	1.00	5.00
Threat of war	117	4.13	0.92	1.00	5.00

In the group of job resources, the study included four sources of support for school principals (Table 4). Principals declared their use the support of school employees to the greatest extent (*M* = 5.01, SD = 0.80) and the support of other principals (*M* = 4.21, SD = 1.10), and to a lesser extent the help of the educational system institutions (*M* = 3.63, SD = 1.08) and using support via social media (*M* = 2.62, SD = 1.52).

**Table 4:** Descriptive statistics for the job resources.

**Job resources**	**N**	**Mean**	**SD**	**Min**.	**Max**.
Support of school employees	116	5.01	0.80	1.00	6.00
Support of other principals	117	4.21	1.10	1.00	6.00
Help of the educational system institutions	117	3.63	1.08	1.00	6.00
Support via social media	117	2.62	1.52	1.00	6.00

A multiple regression analysis was conducted to examine the influence of various factors on burnout levels. The overall model was statistically significant (*F* = 4.794, SE = 0.266, *R*^2^ = 0.21), indicating that it explained 21% of the variance in burnout. The strongest predictor was changes in the educational system (*B* = 2.358, β = 0.310, *t* = 3.467, *p* < 0.001), suggesting that a more negative perception of these changes was associated with higher burnout levels. Another significant protective factor was institutional support from the educational system (*B* = -1.834, β = -0.272, *t* = -2.851, *p* < 0.01), which was associated with lower levels of burnout. Additionally, social media support (*B* = 0.867, β = 0.181, *t* = 2.004, *p* < 0.05) showed a significant positive association, suggesting that more frequent use of social media support may be linked to higher burnout levels. The remaining variables, including perceptions of climate change, economic crisis, threat of war, and support from school staff and other principals, were not significantly associated with burnout levels (*p* > 0.05; Table 5).

**Table 5:** Regression results for predictors of burnout.

**Predictor**	**B**	**SE**	**β**	**t**	**F**	**R^2^**	**ΔR^2^**
**Burnout**					4.794^***^	0.266	0.21
Changes in the educational system	2.358	0.680	0.310	3.467^***^			
Climate change	-1.004	0.719	-0.158	-1.397			
Economic crisis	0.035	0.970	0.004	0.036			
Threat of war	1.321	0.915	0.166	1.445			
Support of school employees	0.189	0.833	0.020	0.226			
Support of other principals	0.963	0.630	0.145	1.530			
Help of educational institutions	-1.834	0.643	-0.272	-2.851^**^			
Support via social media	0.867	0.433	0.181	2.004^*^			

***p < 0.001, **p < 0.01, *p < 0.05.

A multiple regression analysis was conducted to examine the influence of various factors on exhaustion levels. The overall model was statistically significant (*F* = 4.291, SE = 0.245, *R*^2^ = 0.188), explaining 18.8% of the variance in exhaustion. The strongest predictor was changes in the educational system (*B* = 0.163, β = 0.288, *t* = 3.170, *p* < 0.01), indicating that a more negative assessment of these changes was associated with higher exhaustion levels. Similarly, support from the educational system (*B* = -0.137, β = -0.273, *t* = -2.820, *p* < 0.01) was significantly associated with lower levels of exhaustion. Additionally, support from other principals (*B* = 0.096, β = 0.193, *t* = 2.012, *p* < 0.05) showed a positive association, suggesting that more frequent professional peer support may be linked to higher exhaustion levels. The remaining variables, including perceptions of climate change, economic crisis, threat of war, support from school staff, and social media support, were not significantly associated with exhaustion (*p* > 0.05; Table 6).

**Table 6:** Regression results for predictors of exhaustion.

**Predictor**	**B**	**SE**	**β**	**t**	**F**	**R^2^**	**ΔR^2^**
**Exhaustion**					4.291^***^	0.245	0.188
Changes in the educational system	0.163	0.051	0.288	3.170^**^			
Climate change	-0.034	0.054	-0.072	-0.631			
Economic crisis	0.041	0.073	0.068	0.556			
Threat of war	0.063	0.069	0.106	0.914			
Support of school employees	0.029	0.063	0.043	0.467			
Support of other principals	0.096	0.048	0.193	2.012^*^			
Help of educational institutions	-0.137	0.049	-0.273	-2.820^**^			
Support via social media	0.047	0.033	0.131	1.423			

***p < 0.001, **p < 0.01, *p < 0.05.

A multiple regression analysis was conducted to examine the influence of various factors on professional disengagement. The overall model was statistically significant (*F* = 4.455, SE = 0.252, *R*^2^ = 0.195), explaining 19.5% of the variance in professional disengagement. The strongest predictor was the perception of changes in the educational system (*B* = 0.132, β = 0.290, *t* = 3.212, *p* < 0.01), indicating that a more negative assessment of these changes was associated with higher levels of professional disengagement. Additionally, support from educational system institutions (*B* =−0.092, β = -0.229, *t* = -2.373, *p* < 0.05) was significantly associated with lower levels of disengagement. Climate change was also a significant predictor (*B* = -0.091, β = -0.240, *t* = -2.107, *p* < 0.05), suggesting that increased concern about climate change correlated with lower professional disengagement. Furthermore, social media support (*B* = 0.062, β = 0.217, *t* = 2.372, *p* < 0.05) had a positive effect, indicating that more frequent use of social media support may be linked to higher levels of professional disengagement. The threat of war approached statistical significance (*B* = 0.102, β = 0.214, *t* = 1.850, *p* = 0.07), suggesting a potential association with higher professional disengagement, though further research is needed to confirm this effect. The remaining variables, including the economic crisis, support from school staff, and support from other principals, were not significantly associated with professional disengagement (*p* > 0.05; Table 7).

**Table 7:** Regression results for predictors of disengagement.

**Predictor**	**B**	**SE**	**β**	**t**	**F**	**R^2^**	**ΔR^2^**
**Disengagement**					4.455^***^	0.252	0.195
Changes in the educational system	0.132	0.041	0.290	3.212^**^			
Climate change	-0.091	0.043	-0.240	-2.107^*^			
Economic crisis	-0.036	0.058	-0.076	-0.624			
Threat of war	0.102	0.055	0.214	1.850^+^			
Support of school employees	-0.006	0.050	-0.011	-0.117			
Support of other principals	0.024	0.038	0.062	0.646			
Help of educational institutions	-0.092	0.039	-0.229	-2.373^*^			
Support via social media	0.062	0.026	0.217	2.372^*^			

***p < 0.001, **p < 0.01, *p < 0.05, +*p* < 0.10.

## 4 Discussion

Assumptions about job requirements consist of various physical, social and organizational aspects of work that need to be taken into account in the work performed. The theory of organizational stress also indicates factors that have an impact from outside the organization (referred to as extra-organizational) but still have an impact on work performance. The obtained results confirm that in the study of burnout, in addition to intra-organizational factors related to work demands, stressors with a broader, more universal meaning should also be taken into account. They may also negatively impact or accelerate, professional burnout.

Among the non-organizational stress factors examined in this study that may influence burnout among school principals, the perception of changes in the education system emerged as a significant predictor. The remaining stressors factors (climate change, economic crisis, and the threat of war) showed no significant relationship.

The intensity of this stressor is associated with an increase in perceived burnout. The perception of changes in the education system negatively associated with overall level of burnout as well as both of its dimensions. This means that as criticism of these changes increases, levels of exhaustion and disengagement also rise. This may mean that the observed direction of changes in the education system creates work requirements that are perceived as difficult to reconcile with what principals consider “good education” (or changes that are needed in education). At the same time, principals do not have adequate resources to meet those needs or to cope with challenges. They urgently need, e.g., specialized training in change management and stress resilience, mentorship networks of experienced education leaders, and access to professional psychological services.

The perception of the threat of economic crisis, climate change, and war did not emerge as significant predictors of burnout. However, these factors may play an indirect role, mediated by a generalized level of anxiety (42, 43). Testing the mediating role of anxiety in the relationship between the perception of extra-organizational stressors and burnout requires further research.

The second aspect considered in the study was the availability of resources that could mitigate the effects of job demands. One of the fundamental resources that a school principal can develop and/or utilize is support, which may come from colleagues within the organization, other principals, or institutions within the education system. The results indicate that principals most frequently rely on support from school employees. However, this resource is not significantly related to the overall level of professional burnout or any of its dimensions.

The lack of a relationship between professional burnout and the frequency of seeking support from school colleagues may be considered surprising. This finding appears to highlight the loneliness of principals—they engage with others but remain solely responsible for their decisions. Ultimately, they must bear the consequences of their choices alone. These results align with studies on the relationship between professional burnout among university rectors and their use of distributed leadership, which suggest that seeking support from colleagues does not necessarily protect against the emotional and psychological demands of the role (44).

On the other hand, a less frequently used form of support—seeking help from educational institutions—serves as a protective factor against burnout. This suggests that utilizing the support of educational institutions reduces overall burnout levels and positively impacts both its dimensions by decreasing exhaustion and disengagement.

More frequent use of social media support predicts an increase in overall burnout and disengagement. However, this observed pattern may be an artifact: a lack of resources necessary to cope with challenges may lead individuals to seek support from peers in similar roles, despite not having direct personal connections. In this sense, turning to social media for support may act as a “last resort” and indicate a sense of helplessness. Importantly, this association could reflect a reactive coping behavior rather than social media support itself being a direct risk factor. Conversely, such activities may not necessarily enhance the effectiveness of the available resources. Further research is needed to clarify the role of social media support and distinguish cause from coping response.

In conclusion, perceptions of changes in the education system were significantly associated with higher levels of professional burnout, whereas support from educational system institutions was significantly associated with lower burnout levels among school principals.

## 5 Limitation of the study

The study sheds light on the relationship between burnout and extra-organizational factors and job resources. Nevertheless, it is not free from several limitations. Firstly, the study used selected extra-organizational stressors, which do not capture the full diversity of potential challenges; potentially, subsequent research may include factors related to life crises, family problems, social status of the role performed, or natural disasters (e.g., loss of property, involvement in rescue operations). Secondly, we also acknowledge that the *ad hoc* questionnaire used to assess extra-organizational stressors has not undergone formal psychometric validation, which may limit the precision of these measures without detracting from the exploratory insights of this study. Thirdly, the sample is limited to school principals in a large city, and assessments of extra-organizational stressors and work resources may differ in smaller towns or villages. Fourthly, the social climate during data collection—conducted in the period preceding the parliamentary elections in Poland—may have introduced additional tension related to uncertainty about the future, intensified debates on educational reforms, and heightened concerns over the economic and geopolitical situation.

## 6 Conclusions

The conducted research confirmed a significant relationship between extra-organizational factors rarely used in research and burnout. A negative assessment of changes in the education system and the perception of an increased threat of economic crisis correlates with a higher level of burnout. Varied relationship between job resources (use of assistance) and burnout was observed. Using the help of educational institutions is associated with a lower level of burnout, while using the help of other principals correlates with a higher level of work exhaustion. The correlation between using help from school employees and using help via social media with both burnout scales turned out to be non-significant.

## Data Availability

The raw data supporting the conclusions of this article will be made available by the authors, without undue reservation.
